# Trauma-Induced Nanohydroxyapatite Deposition in Skeletal Muscle is Sufficient to Drive Heterotopic Ossification

**DOI:** 10.1007/s00223-018-0502-5

**Published:** 2018-12-04

**Authors:** Stephanie N. Moore-Lotridge, Qiaoli Li, Breanne H. Y. Gibson, Joseph T. Martin, Gregory D. Hawley, Thomas H. Arnold, Masanori Saito, Sami Tannouri, Herbert S. Schwartz, Richard J. Gumina, Justin M. M. Cates, Jouni Uitto, Jonathan G. Schoenecker

**Affiliations:** 1 Department of Orthopaedics and Rehabilitation, Vanderbilt University Medical Center, 1215 21st Ave. South, Suite 4200 MCE, South Tower, Nashville, TN 37232 USA; 2 Department of Pathology Microbiology and Immunology, Vanderbilt University Medical Center, 1161 21st Ave. South, Nashville, TN 37232 USA; 3 Department of Pediatrics, Vanderbilt University Medical Center, 4202 Doctor’s Office Tower, 2200 Children’s Way, Nashville, TN 37232 USA; 4 Division of Cardiovascular Medicine, Vanderbilt University Medical Center, 2220 Pierce Ave, Preston Research Building, Nashville, TN 37232 USA; 5 Department of Pharmacology, Vanderbilt University, 2200 Pierce Ave, Robinson Research Building, Nashville, TN 37232 USA; 6 College of Arts and Science, Vanderbilt University, 301 Kirkland Hall, Nashville, TN 37240 USA; 7 Department of Dermatology and Cutaneous Biology, Sidney Kimmel Medical College, Thomas Jefferson University, 233 South Tenth Street, Bluemle Life Sciences Building, Room 450, Philadelphia, PA 19107 USA; 8 2200 Pierce Ave, Robinson Research Building, Rm 454, Nashville, TN 37232 USA; 9 Present Address: University of Nebraska Medical Center, Omaha, NE USA

**Keywords:** Heterotopic ossification, Nanohydroxyapatite, Dystrophic calcification, Skeletal muscle injury, Abcc6

## Abstract

Heterotopic ossification (HO), or the pathologic formation of bone within soft tissues, is a significant complication following severe injuries as it impairs joint motion and function leading to loss of the ability to perform activities of daily living and pain. While soft tissue injury is a prerequisite of developing HO, the exact molecular pathology leading to trauma-induced HO remains unknown. Through prior investigations aimed at identifying the causative factors of HO, it has been suggested that additional predisposing factors that favor ossification within the injured soft tissues environment are required. Considering that chondrocytes and osteoblasts initiate physiologic bone formation by depositing nanohydroxyapatite crystal into their extracellular environment, we investigated the hypothesis that deposition of nanohydroxyapatite within damaged skeletal muscle is likewise sufficient to predispose skeletal muscle to HO. Using a murine model genetically predisposed to nanohydroxyapatite deposition (ABCC6-deficient mice), we observed that following a focal muscle injury, nanohydroxyapatite was robustly deposited in a gene-dependent manner, yet resolved via macrophage-mediated regression over 28 days post injury. However, if macrophage-mediated regression was inhibited, we observed persistent nanohydroxyapatite that was sufficient to drive the formation of HO in 4/5 mice examined. Together, these results revealed a new paradigm by suggesting the persistent nanohydroxyapatite, referred to clinically as dystrophic calcification, and HO may be stages of a pathologic continuum, and not discrete events. As such, if confirmed clinically, these findings support the use of early therapeutic interventions aimed at preventing nanohydroxyapatite as a strategy to evade HO formation.

**Electronic supplementary material:**

The online version of this article (10.1007/s00223-018-0502-5) contains supplementary material, which is available to authorized users.

## Introduction

Heterotopic ossification (HO) is the formation of bone within injured soft tissues such as skeletal muscle or tendons. In addition to imposing pain from chronic inflammation and tissue deformation, peri-articular HO restricts joint mobility and limb function thus precluding activities of daily living [1, 2]. While reports vary, HO is a significant problem in the civilian population, particularly following severe injury. For example, up to 25% of traumatic acetabular fractures, 20% of spinal cord injuries, and 11% of brain injuries have been reported to be complicated by HO development [3–5]. The incidence of HO dramatically rises in military-related injuries, such that HO affected up to 65% of the severe wartime extremity injuries during the Afghanistan and Iraqi conflicts [6]. Although of great significance to both the civilian and military population, the pathophysiology of HO remains poorly understood and, consequently, current treatments are suboptimal. Thus, studies aimed at defining the pathophysiology of HO to develop novel therapeutic strategies, especially following severe injuries in military populations, are essential.

The majority of soft tissue injuries experienced by patients do not form HO, rather they repair fully to the original tissues form. Failure of tissue repair typically results in fibrosis, devoid of bone. Yet, following traumatic injuries, a subset of patients develops HO. As an explanation to this phenomenon, Chalmers et al. proposed in 1975 that soft tissue injury was a prerequisite of developing HO, and additional predisposing factors that favor ossification within the injured soft tissues environment were available to support bone formation [7]. In support of this theory, it was identified in 2006 that patients and animals with fibrodysplasia ossificans progressiva (FOP), a genetically driven form of HO, possess gain-of-function mutations in the Type 1 bone morphogenic protein (BMP) receptor, which favors cellular transformation within injured soft tissue towards an osteoblast lineage [8, 9]. Although these mutations have been demonstrated to be the causative factor in FOP, genome wide association studies have failed to correlate mutations in the BMP signaling pathways with the greater majority of trauma-induced HO [10], suggesting that, in non-FOP cases, alternative predisposing pro-ossification factors may be responsible.

The primary mechanism by which chondrocytes and osteoblasts, two pro-ossification cells, initiate bone mineralization is by stimulating the formation and accumulation of nanohydroxyapatite crystals upon collagen X and collagen I within their extracellular environment [11]. Importantly, calcium and phosphate circulate at near-saturating concentrations [12] thereby favoring the formation of nanohydroxyapatite by these pro-ossification cells [13]. While these ionic concentrations are ideal for maintaining bone integrity, soft tissue and homeostatic environments are simultaneously exposed to these saturating conditions; thus if a nucleating matrix is available, crystal formation will progress [12, 13].

Given the pathologic implications of hardened or mineralized soft tissues, the body possesses a myriad of factors that either prevent or dissolve nanohydroxyapatite [13–29]. One of the most well-described soft tissue protection mechanisms is the production of pyrophosphate (PPi), a potentate anti-mineralization molecule, produced primarily from the cleavage of adenosine triphosphate (ATP) [30, 31]. Thus, in accordance with Chalmers’ theory [7], we surmise that if a predisposing ossification factor such as nanohydroxyapatite was not regulated within soft tissues, its deposition could mimic the bone environment and favor ossification. From this scientific premise, we hypothesized that, as an alternative to a gain-of-function of an ossification gene (as seen in FOP), a loss-of-function mutation in the molecular mechanisms that protect soft tissues from nanohydroxyapatite formation may also be sufficient to support HO following soft tissue injury.

To test this hypothesis, we investigated the fate of skeletal muscle following injury in a murine model of a rare calcification disorder, pseudoxanthoma elasticum (PXE, incidence of 1:50,000). PXE occurs due to biallelic null mutations primarily in *Abcc6* (ATP binding cassette sub family C, member 6), an ATP transporter produced in the liver responsible for moving ATP from the intracellular space to the extracellular environment where it is cleaved by ectonucleotide pyrophosphatase/phosphodiesterase 1 (ENPP1) to produce AMP and PPi, a potent inhibitor of nanohydroxyapatite formation. As such, patients and animals with PXE have diminished PPi and develop progressive deposits of nanohydroxyapatite within the skin, cardiovascular system, and retinas [16, 30–35]. Yet, the role of *Abcc6* in preventing nanohydroxyapatite and HO in skeletal muscle following injury is unknown. Here, we investigated two related hypotheses: (1) to determine if a loss of *Abcc6* promotes nanohydroxyapatite deposition within injured muscle following injury and (2) if so, whether nanohydroxyapatite deposition in the injured muscle environment is sufficient to promote HO.

### Materials and Methods

#### Operational Definitions of Soft Tissue Calcification

The following operational definitions will be utilized throughout in reference to various forms of calcification within skeletal muscle, delineated by their unique morphological properties. (1) Dystrophic calcification will be used broadly to describe deposits of amorphous calcium phosphate crystals within soft tissues. (2) Nanohydroxyapatite is a sub-classification of dystrophic calcification that will be utilized to describe calcium phosphate deposits that are both (i) within the nanometer range and (ii) the molecular composition of hydroxyapatite. (3) HO is defined as bone formation in soft tissue, characterized by the presence of woven bone, hematopoietic marrow, and/or the presence of rimming osteoblasts.

#### Murine Model of Skeletal Muscle Calcification

##### Animal Husbandry

*Abcc6*^+/+^, *Abcc6*^+/−^, and *Abcc6*^−/−^ mice were housed within Vanderbilt University Medical Center under a 12-h light/dark cycle with free access to food and water. All studies were conducted in 6-week-old animals on a C57BL/6J background fed a standard chow diet. Equal numbers of male and female animals were included in each cohort.

#### Murine Cardiotoxin-Induced Muscle Injury Model

Following adequate anesthesia with isoflurane, focal muscle injury was induced via intramuscular injection of 40 µL of 10 nM cardiotoxin (Accurate Chemical and Scientific Corp, Westbury, NY) into the posterior compartment of the lower leg using a lateral approach with a 28.5 g, 0.5 mL, insulin syringe as previously described [36, 37]. Both the right and left posterior compartment muscles of the lower extremity were injured and analyzed by radiography for the presence of skeletal muscle calcification.

### Output Analysis and Quantification of Skeletal Muscle Calcification

#### Radiographic Analysis

Beginning 7 days post injury (DPI) and continuing through sacrifice, digital radiographs (Faxitron, Tucson, AZ) of the lower extremity were acquired. Following adequate anesthesia, mice were placed in the prone position with hips in abduction, allowing for external rotation of the leg by placing the tibia in a reproducible lateral position. Single plane lateral radiographic images were collected at an exposure of 4 s at 35 kV and saved as a DICOM (.dcm) files for image processing and quantification.

#### Soft Tissue Calcification Scoring System (STiCSS) Quantification

The STiCSS is a previously validated ordinal grading system developed by our lab to quantify the extent of soft tissue calcification within the posterior compartment muscle of the lower extremity longitudinally by serial radiographic analysis [37]. Briefly, the operational definitions of each score are based on the percentage area of soft tissue calcification observed in the posterior compartment of the lower extremity: 0 (0%), 1 (1–25%), 2 (25–49%), 3 (50–75%), and 4 (> 75%).

#### Micro CT Analysis

µCT images of the injured hind limbs were acquired following sacrifice at 55 kVp, 145 µA, 200 ms integration, 500 projections per 180° rotation, with a 20 µm isotropic voxel size (µCT40, Scanco Medical AG, Bassersdorf, Switzerland). After reconstruction, a volume of interest comprising the region of soft tissue calcification within the posterior compartment of the lower extremity was selected as previously described [36]. Mineralized tissue within the volume of interest was segmented from soft tissue using a threshold of 220/1000 (or 450.7 mgHA/cm^3^), a Gaussian noise filter of 0.2, and support of 1.

#### Histological Analysis

Injured hind limbs were fixed in 10% neutral-buffered formalin for 24–72 h. All samples were processed in graded series of ethanol, cleared, and embedded in paraffin prior to sectioning. 6-µm sections were cut and stained as described below.

##### Hematoxylin and Eosin (H/E) Staining

Deparaffinized sections were stained in Gills 3 hematoxylin solution for 5 min. Slides were rinsed in tap water for 10 min followed by eosin staining for 2 min. Slides were then dehydrated and cleared in xylene before mounting with Permount. Histological quantification of skeletal muscle damage was assessed by light microscopy at × 200 magnification (Axio imager a1, ZEISS; Oberkochen, Germany) as previously described [36]. At least 3 mice were analyzed per group, with > 2 sections per mouse and > 4 images per section (i.e., minimum of 24 images per group). Briefly, skeletal muscle damage was evaluated in a blinded manner by counting (1) ‘damaged sarcomeres’ identified by hypereosinophilic sarcoplasm and centrally located nuclei, (2) ‘calcified sarcomeres’ identified by dense basophilic staining for hematoxylin, and (3) histologically normal sarcomeres. These groups were then expressed as a percentage of total muscle fibers within × 200 magnification field.

##### Von Kossa Staining for Calcification

Deparaffinized sections were rinsed with distilled water and exposed to 1% AgNO_3_ solution under UV light for 30 min. Slides were counterstained with Fast Green for 5 min, dehydrated, and cleared in xylene before mounting with Permount.

##### Martius Scarlet Blue (MSB) Staining

Following deparaffinization, staining was performed per standard protocols to assess for fibrin and collagen deposition within damaged tissues. Briefly, deparaffinized sections were rinsed with tap water and stained with Wiegert’s Hematoxylin for 5 min. Slides were then rinsed, differentiated in 1% acid alcohol for 15 s, rinsed again in tap water, and cleared in several changes of 95% ethanol. Next, slides were placed into working Martius yellow solution for 2 min, rinsed, and stained with Crystal Ponceau 6R for 10 min. Slides were then differentiated in 15 phosphotungstic acid for 5 min, washed, and finally stained with methyl blue solution for 5 min prior to dehydration through graded ethanol, clearing with xylene, and mounting with Permount.

##### Immunohistochemical (IHC) Staining of F4/80+ Cells

IHC for F4/80+ cells indicative of monocyte lineage was performed per standard protocols in a core facility (Vanderbilt Translational Pathology Shared Resource; http://www.mc.vanderbilt.edu/tpsr). % Area of positive F4/80 staining was quantified by ImageJ through the use of the IHC toolkit freely provided.

#### Energy Dispersive X-Ray Analysis

Sections of muscle were analyzed using energy dispersive X-ray (EDS) analysis and topographic mapping. Paraffin sections were mounted onto carbon carriers, imaged, and analyzed for elemental composition with a FEI 600 Quanta FEG scanning electron microscope (FEI Company, Eindhoven, The Netherlands) fitted with an Octane Super SDD EDS detector (EDAX, Sandy, UT, USA). X-ray topographic (RADAR) maps of calcium and phosphorus were acquired using Spirit software version 1.07.05 (Princeton Gamma-Tech, Rocky Hill, NJ, USA). EDS spectra and topographic maps were collected for 60.8 and 717.5 s (80 frames), respectively.

#### Macrophage Depletion

Depletion of macrophages was accomplished through intravenous administration of 200 µL of clodronate or PBS-filled (control) liposomes (Liposoma, Amsterdam, The Netherlands) with a 28.5-g, 0.5-mL, insulin syringe weekly beginning at the time of injury until sacrifice [38, 39].

### Transition Electron Microscopy to Visualize Macrophage-Mediated Dystrophic Calcification Regression

Specimens were processed for transition electron microscopy (TEM) and imaged in the Vanderbilt cell imaging shared resource-research electron microscopy facility.

#### Embedding

Samples were fixed in 2.5% glutaraldehyde in 0.1 M cacodylate buffer, Ph 7.4, at room temperature (RT) for 1 h and then transferred to 4 °C, overnight. Samples were washed in 0.1 M cacodylate buffer, incubated for 1 h in 1% osmium tetraoxide at RT, and then washed with 0.1 M cacodylate buffer. Subsequently, samples were dehydrated through a graded ethanol series and then 3 exchanges of 100% ethanol. Next, samples were incubated for 5 min in 100% ethanol and propylene oxide (PO) followed by 2 exchanges of pure PO. Samples were then infiltrated with 25% Epon 812 resin and 75% PO for 30 min at RT. Next, samples were infiltrated with Epon 812 resin and PO [1:1] for 1 h at RT and then overnight at RT. Next day, the samples went through a [3:1] (resin: PO) exchange for 3–4 h, and then incubated with pure epoxy resin overnight. Samples were then incubated in two more changes of pure epoxy resin and then allowed to polymerize at 60 °C for 48 h.

#### Sectioning and Imaging

70–80-nm ultra-thin sections were cut and collected on 200-mesh copper grids and post-stained with 2% uranyl acetate and then with Reynold’s lead citrate. Samples were subsequently imaged on the Philips/FEI Tecnai T12 electron microscope at various magnifications.

### Statistics and Data Handling

STiCSS scores between the indicated cohorts were compared using the non-parametric Mann–Whitney or Kruskal–Wallis test with correction for multiple comparisons (Dunn’s multiple comparisons test) as previously validated [37]. Error bars represent median with interquartile range. Quantification and statistical analysis of skeletal muscle healing was assessed with a Kruskal–Wallis test with correction for multiple comparisons. *p* values reported are corrected for multiple comparisons. Statistical analyses were performed in GraphPad Prism (v6, GraphPad Software, La Jolla, CA) with *α* = 0.05, and two-sided testing was applied. Number of mice (*N*) per group and number of limbs assessed (*n*) are reported in the designated figure or figure legend.

#### Sample Size

Calculation for sample size was based upon previously published investigations [36, 37]. Previously, we determined that 3 mice per groups were necessary to detect a 100% change in soft tissue calcification quantified by radiographic analysis. Therefore, all studies were conducted with an excess of 3 mice per group.

#### Data Collection and Inclusion

Data from all animal experiments were collected at either 7, 14, or 28 days post injury as indicated within the figures or figure legends. During these experiments, no animals experience adverse consequences necessitating their removal from the study; therefore, no additional endpoints were assessed. The selected endpoints were previously established, prior to this investigation, with the approval of our animal use protocols. All radiographic data collected were quantified and included within this article. No animals were excluded from the study. Histological results were conducted on a minimum of 3 mice per group, with the image included representing the mean response observed.

#### Randomization and Blinding

Once genotyped, all male and female mice from a single litter were randomly assigned to an experimental or treatment group. Mice of different genotypes or treatment group were mixed within each cage. Individuals quantifying weekly radiographic images for the amount of soft tissue calcification were blinded to the genotype or experimental group of the animals. Furthermore, histologic slides were blinded prior to quantification of skeletal muscle regeneration. For studies conducted on high phosphate diet, mice were randomly assigned to each diet groups at the time of weaning. However due to the clear color difference in food, we were not able to blind the diet groups to the investigators obtaining weekly radiographs. Yet, radiographs were quantified by a separate party in a blinded manner.

## Results

### Loss of ABCC6 Predisposes Skeletal Muscle to Nanohydroxyapatite Deposition Following Injury

**Fig. 1:**
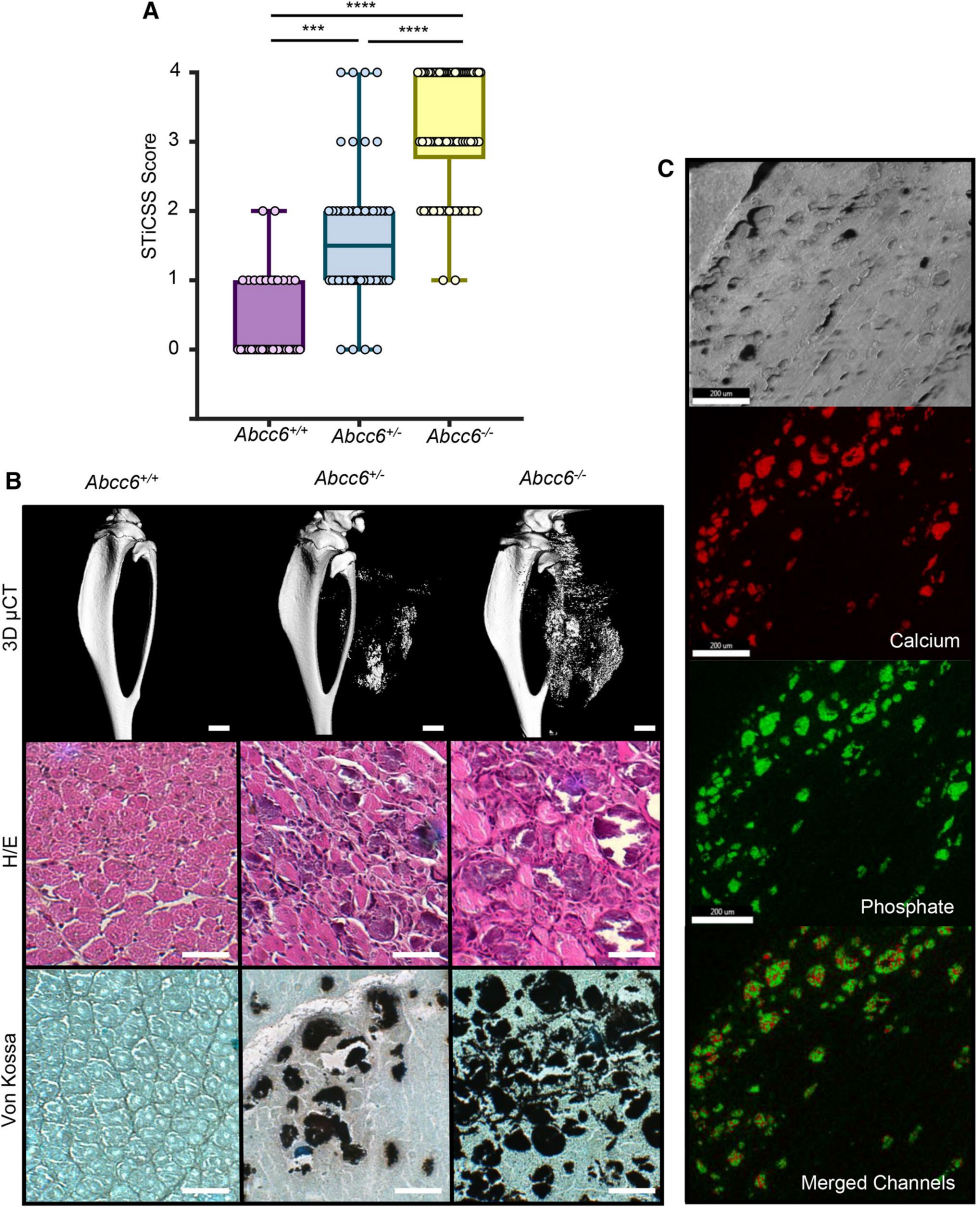
Loss of ABCC6 predisposes skeletal muscle to nanohydroxyapatite deposition at 7DPI. **a** WT (*Abcc6*^+/+^), heterozygous (*Abcc6*^+/−^), and homozygous (*Abcc6*^−/−^) mice were assessed for calcification at the site of skeletal muscle injury by radiographic analysis and subsequent STiCSS quantification at 7 DPI. See Table 2 for detailed analysis of the genotypes and N. ***p < 0.001; ****p < 0.0001. Statistical analysis between groups was performed using a non-parametric Mann–Whitney test. **b** Representative 3D µCT reconstructions and histological analysis of skeletal muscle calcification within the injured gastrocnemius and soleus muscles at 7 DPI. Scale bar represents 100 µm. *n* ≥ 3 mice per genotype. Positive Von Kossa staining, noted by the black deposits, indicates calcium deposition within the damaged skeletal muscle. **c** Energy dispersive X-ray (EDS) analysis of dystrophic calcification nodules within damaged ABCC6-deficient skeletal muscle at 14 DPI. Topographic mapping demonstrated marked co-localization of calcium and phosphate with an average calcium/phosphate atomic ratio of 1.67 ± 0.2, indicative of hydroxyapatite. Analysis was conducted following random sampling of 5 distinct spots per tissue section. Scale bar represents 200 µM, thereby indicating nanohydroxyapatite

Utilizing mice with either partial (*Abcc6*^+/−^) or complete (*Abcc6*^−/−^) genetic reduction of ABCC6, we observed a gene-dependent predisposition for dystrophic calcification within the injured skeletal muscle at 7 DPI as measured by radiographic analysis and µCT (Fig. 1a and b; Table 1). Further analysis by energy dispersive X-ray (EDS) and histologic analysis demonstrated that the dystrophic calcification present within damaged skeletal muscle was in the nanometer range and possessed both inorganic calcium and phosphate with an average calcium/phosphate atomic ratio of 1.67 ± 0.2, indicative of nanohydroxyapatite (Fig. 1c). Together, these results demonstrate that loss of ABCC6 is sufficient to predispose skeletal muscle, like other soft tissues, to the deposition of nanohydroxyapatite following injury.

**Table 1:** Quantification of skeletal muscle calcification in ABCC6-deficient mice at 7DPI

STiCSS score	ABCC6^+/+^		ABCC6^+/−^		ABCC6^−/−^	
	*N*	%	*N*	%	*N*	%
0	43	67.2	4	7.7	0	0
1	20	31.3	22	42.3	2	2.3
2	1	1.5	18	34.6	19	22.1
3	0	0	4	7.7	23	26.7
4	0	0	4	7.7	42	48.9
*N* (*n*)	64 (32 mice)		52 (26 mice)		86 (43 mice)	
Median	0.0		1.5		3.0	

*N* represents total number of individual samples analyzed, with the left and right leg acting as individual samples. *n* represents total number of mice per group. Equal number of male and females was used in each genotype

**Fig. 2:**
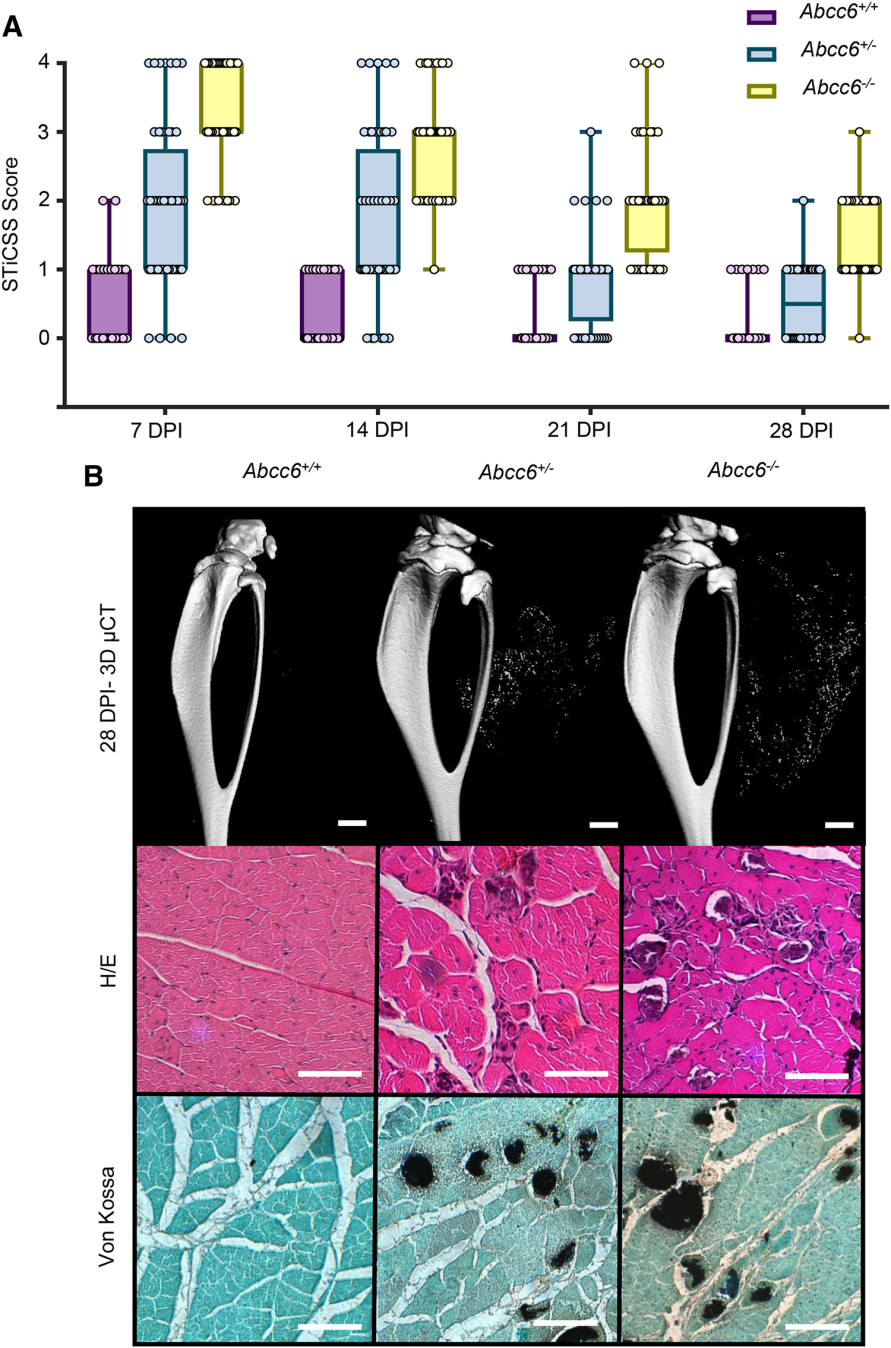
Nanohydroxyapatite deposition in ABCC6-Deficient Mice is Degraded Over 28 DPI. Beginning at 7DPI, *Abcc6*^+/+^, *Abcc6*^+/−^, or *Abcc6*^−/−^ animals were **a** assessed weekly by radiographic analysis through 28 DPI and quantified by the STiCCS, to reveal progressive resolution of nanohydroxyapatite from damaged skeletal muscle. **b** 3D µCT and histologic analysis at 28 DPI demonstrates reduced nanohydroxyapatite deposition compared to results seen in 7 DPI. H/E staining was utilized to assess sarcomere morphology and regeneration quantified in Table 2, and Von Kossa Staining was used to visualize calcification. Scale bar represents 100 µm. *n* ≥ 3 mice per genotype

**Table 2:** Histological quantification of skeletal muscle calcification and regeneration at 7 and 28 DPI

Genotype (timepoint)		*N*	% Histologically normal sarcomeres	Regenerating sarcomeres	Calcified or damaged sarcomeres
ABCC6^+/+^	7 DPI	8	26.53 ± 15.31^a^	72.40 ± 15.30	1.07 ± 1.92
ABCC6^+/−^	7 DPI	8	18.81 ± 11.51^a^	56.92 ± 15.95	24.27 ± 15.24^d,e^
ABCC6^−/−^	7 DPI	11	18.50 ± 13.71^a^	43.44 ± 11.07^c^	38.06 ± 15.84^f^
ABCC6^+/+^	28 DPI	6	42.20 ± 17.67^b^	57.45 ± 18.18	0.35 ± 0.86^g^
ABCC6^+/−^	28 DPI	13	39.37 ± 16.81^b^	54.78 ± 16.85	5.82 ± 3.64^h^
ABCC6^−/−^	28 DPI	13	31.47 ± 10.53^b^	51.78 ± 11.78	16.75 ± 4.89^i^

Detailed histological analysis of Abcc6^+/+^, Abcc6^+/−^, and *Abcc6*^−/−^ mice at 7 and 28 DPI. *N* number of individuals analyzed per time point, mixed between males and females. Four sections per mouse and four images per section were analyzed as outline in Materials and Methods section

^a^Non-significant difference between groups, *p* > 0.05

^b^Non-significant difference between groups, *p* > 0.05

^c^Abcc6^+/+^ versus Abcc6^−/−^, **, *p* = 0.001

^d^Abcc6^+/+^ versus Abcc6^+/−^, ***p = 0.0001

^e^Abcc6^+/−^ versus Abcc6^−/−^, *p = 0.028

^f^Abcc6^+/+^ versus Abcc6^−/−^, ****p < 0.0001

^g^Abcc6^+/+^ 7 versus 28 DPI, non-significant difference, *p* > 0.05

^h^Abcc6^+/−^ 7 versus 28 DPI, ***p = 0.0007

^i^Abcc6^−/−^ 7 versus 28 DPI, ****p < 0.0001

#### Nanohydroxyapatite Deposited in Skeletal Muscle Following Injury is Reversible

Given the above findings, we next examined the second hypothesis that nanohydroxyapatite deposited within skeletal muscle following injury would be sufficient to promote HO. Longitudinal radiographic analysis of Abcc6-deficient mice demonstrated robust nanohydroxyapatite depositing within injured tissues at 7 DPI that progressively decreased in both *Abcc6*^+/−^ and *Abcc6*^−/−^ mice over 28 DPI (Fig. 2a). Histological quantification of damaged skeletal muscle at 7 and 28 DPI in *Abcc6*^+/+^, *Abcc6*^+/−^, and *Abcc6*^−/−^ demonstrated comparable initial levels of injury between cohorts, given the comparable percentage of histologically normal sarcomeres, and confirmed the decrease of nanohydroxyapatite in *Abcc6*^+/−^ and *Abcc6*^−/−^ between 7 and 28 DPI (Fig. 2b; Table 2). Taken together, while loss of ABCC6 is sufficient to predispose skeletal muscle to nanohydroxyapatite deposition at 7 DPI, the mineral deposited was progressively removed from the damaged skeletal muscle and therefore was insufficient to promote ossification.

#### Macrophages Mediate Nanohydroxyapatite Resorption from Damaged Skeletal Muscle

Given the progressive decrease in nanohydroxyapatite from damaged skeletal muscle over 28 DPI, we next investigated (1) the mechanisms by which nanohydroxyapatite was degraded and (2) the pathologic consequence of impaired nanohydroxyapatite degradation on skeletal muscle healing.

**Fig. 3:**
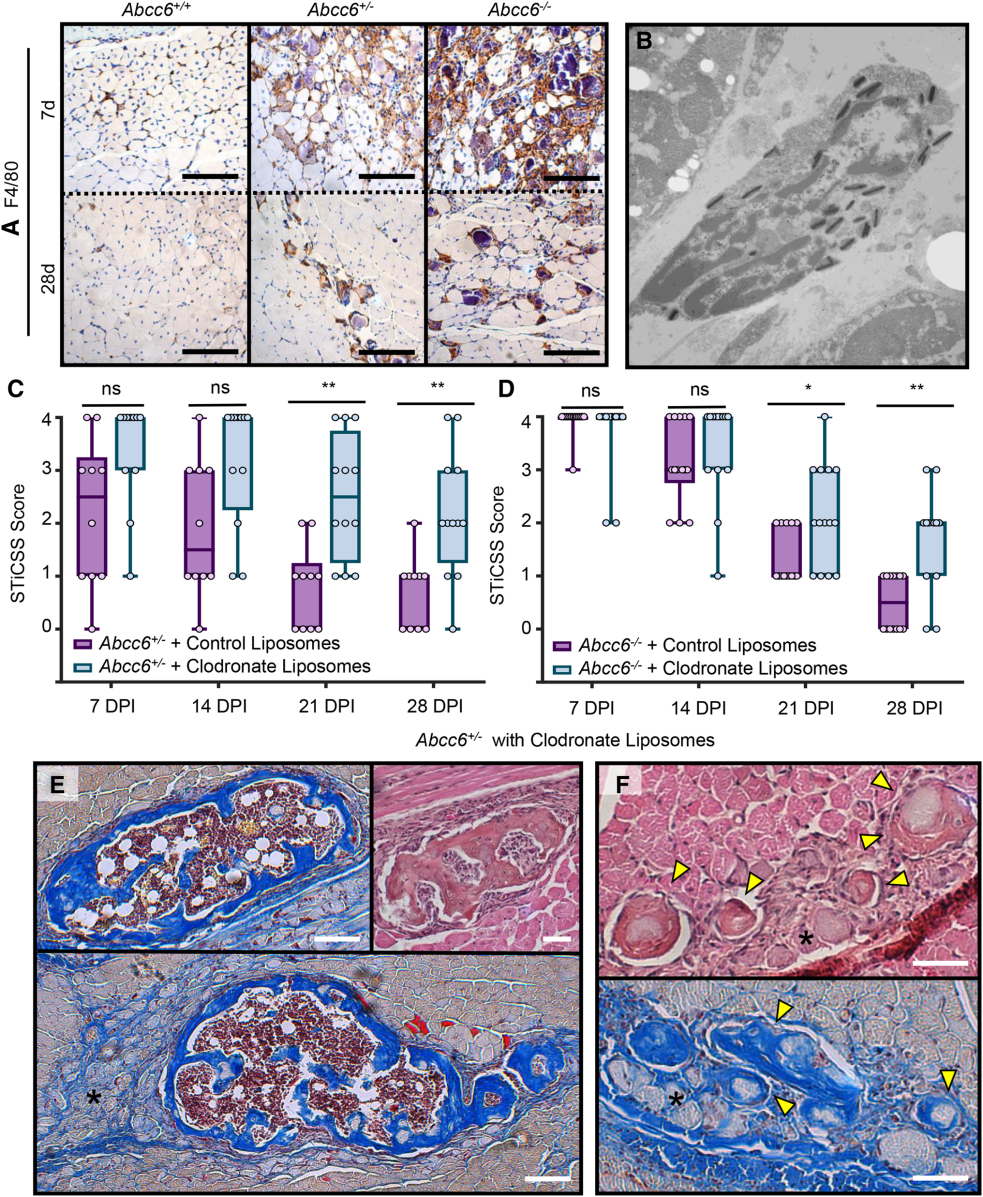
Macrophage-mediated resolution of nanohydroxyapatite prevents maturation to HO. **a** Immunohistochemical stain for F4/80+ cells at 7 and 28 DPI in injure skeletal muscle from *Abcc6*^+/+^, *Abcc6*^+/−^, or *Abcc6*^−/−^ mice. **b** Transition electron microscope image of a macrophage containing phagocytosed nanohydroxyapatite. Scale bar represents 500 nm. Image was obtained from a WT C57BL/6J following CTX injury at 3 days post injury when macrophage infiltration to damaged tissue is greatest [36]. **c** Longitudinal STiCSS analysis of Abcc6^+/−^ treated with either control (PBS) or clodronate-filled liposomes beginning at the time of injury. *N* ≥ 5 mice per group. Nanohydroxyapatite was not observed in * Abcc6*^+/+^ mice treated with either control or clodronate-filled liposomes, *N* ≥ 4 mice per group. Data not shown. **p < 0.01; ***p < 0.001. **d** MSB histological analysis at 28 DPI of *Abcc6*^+/−^ mice treated with control liposomes. **e, f** Histological analysis of *Abcc6*^+/−^ mice treated with clodronate-containing liposomes indicating the presence of mature HO (E, MSB, and H/E) characterized by the presence of woven bone, blue staining (MSB) indicative of collagen deposition, and central hematopoiesis. Regions of persistent nanohydroxyapatite (black asterisks), and regions that appear to be nanohydroxyapatite maturing to ossified lesions (F-Yellow arrows, MSB, and H/E) were also observed. Scale bar represents 100 µm

Previously, macrophages have been suggested to assist in the resorption of calcification in vitro and within the cardiovascular system, yet it is unknown what role macrophage plays in regression of nanohydroxyapatite deposited in skeletal muscle. Here, histological analysis of *Abcc6*^+/−^ and *Abcc6*^−/−^ injured skeletal muscle demonstrated foci of nanohydroxyapatite surrounded by a cellular inflammatory infiltrate consisting of F4/80+ macrophages/monocytes. This infiltrate was present at 7 DPI and persisted through 28 DPI, where it was largely focused around the remaining sites of nanohydroxyapatite (Fig. 3a). When quantified, the % area of positive F4/80 staining increased in a gene-dependently at both 7 and 28 DPI (Suppl Fig. 1), aligning with the gene-dependent levels of calcification observed previously by radiographic and histologic analysis (Figs. 1, 2) Transition electron microscopy of injured skeletal muscle revealed macrophages adjacent to damaged sarcomeres containing dense encapsulated granules, indicative of resorbed nanohydroxyapatite (Fig. 3b). Furthermore, when macrophages were inhibited in either *Abcc6*^+/−^- or *Abcc6*^−/−^-deficient animals via liposome-targeted clodronate administration, we observed significant inhibition of nanohydroxyapatite resorption through 28 DPI compared to control-treated animals (Fig. 3c and d). Taken together, these data suggest that macrophages are present within damaged tissues and are participating in the resorption of nanohydroxyapatite from damaged skeletal muscle.

#### Pathologic Consequence of Impaired Nanohydroxyapatite Resorption

Histological analysis of *Abcc6*^+/−^ tissue at 28 DPI demonstrated that while both cohorts of mice developed nanohydroxyapatite, in mice lacking macrophage-mediated resorption, nanohydroxyapatite was persistent in 5/5 mice and sufficient to support HO in 4/5 mice analyzed, characterized by the presence of woven bone and central hematopoiesis (Fig. 3d and e, Supp Figs. 2 and 3). Detailed assessment of these regions demonstrated small focal areas morphologically akin to HO as well as regions that appear to be nanohydroxyapatite transitioning to ossified lesions (Fig. 3f—yellow arrows). Together, these data suggest that (1) macrophages are an essential cellular mediator, capable of regressing nanohydroxyapatite from damaged skeletal muscle, and (2) if macrophages are inhibited, persistent nanohydroxyapatite within damaged tissues is sufficient to predispose damaged skeletal muscle to HO formation.

## Discussion and Conclusions

**Fig. 4:**
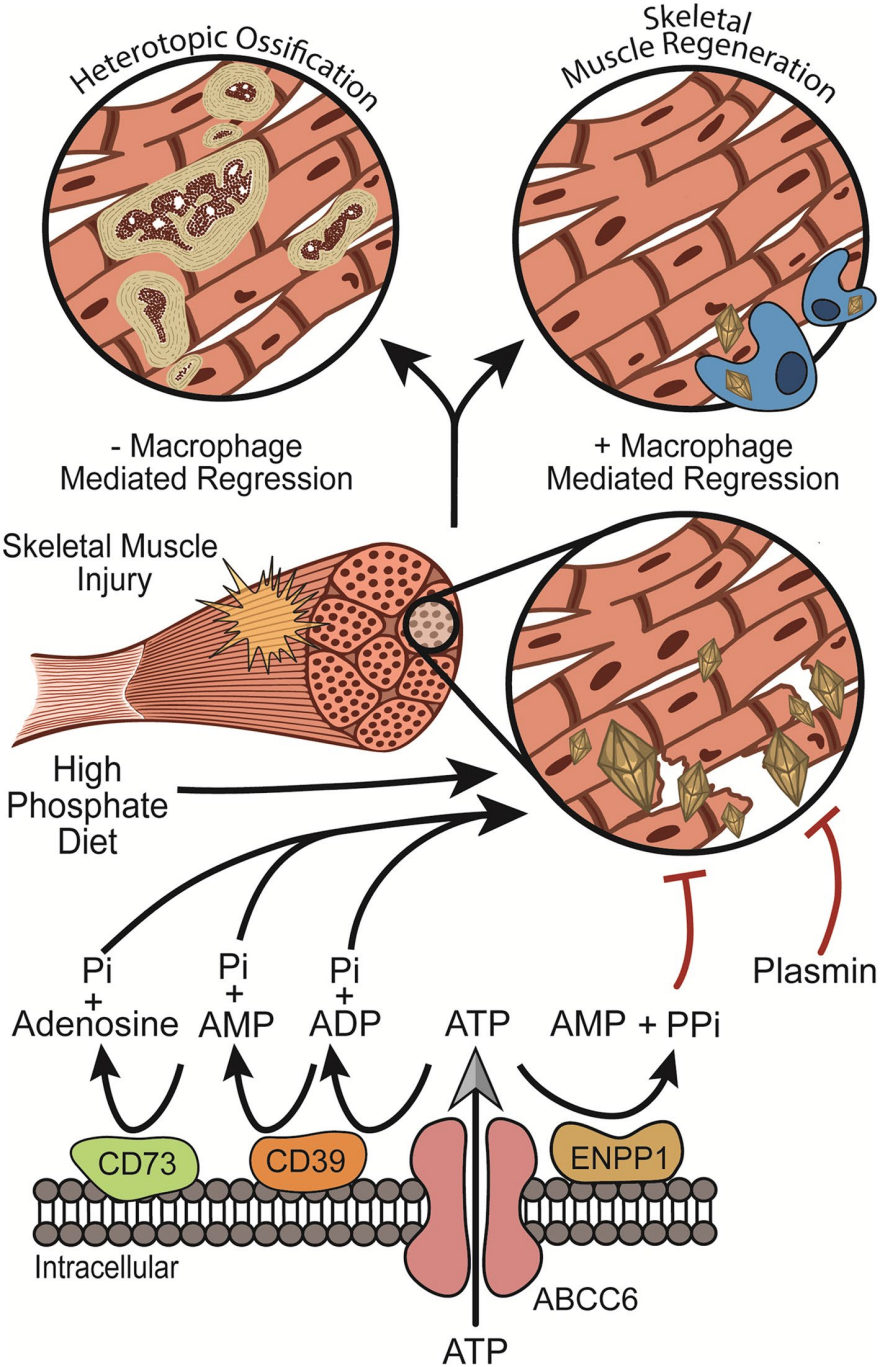
The “Two Hit” mechanism of HO formation: taken together with previous studies [36], our results suggest that nanohydroxyapatite can deposit with in skeletal muscle following injury if one of the skeletal muscle protection mechanisms (i.e., ABCC6 or plasmin) are insufficient. Fortunately, the body possesses a secondary macrophage-mediated clean-up crew to regress nanohydroxyapatite from damaged tissues, thereby resolving the predisposing factors to HO formation. Together, these two lines of defense are critical for preventing nanohydroxyapatite deposition within damaged tissues and its subsequent maturation to HO. These findings suggest a new paradigm for HO formation in which HO can result from insufficient protection against nanohydroxyapatite with a failure of macrophage-mediated regression

Since proposed by Chalmers in 1975 [7], the theory that HO requires a pro-ossification stimulus within an injured soft tissue environment has been maintained. This theory supported the search for gain-of-function mutations in soft tissues, such as those found in the BMP pathway (i.e., FOP), responsible for cellular transformation of soft tissue cells into bone-forming cells. Here, we demonstrate that a loss-of-function mutation in the cellular machinery designed to protect soft tissue from nanohydroxyapatite deposition is an additional mechanism through which HO can form following soft tissue injury. Interestingly, we observed that as a contingency plan to the primary soft tissue protection mechanisms, macrophages efficiently resorbed nanohydroxyapatite immediately prior to myogenesis. In the absence of both synergistic soft tissue protection mechanisms (loss of both Abcc6 and macrophage resorption), HO ensued. These findings support a potential additional paradigm in HO which can result from insufficient protection against nanohydroxyapatite with a subsequent failure of macrophage-mediated resorption (Fig. 4).

Nanohydroxyapatite deposition within soft tissues can occur from a variety of risk factors, including both genetic and environmental (i.e., high phosphate diet) sources [13–29]. Here, we observed for the first time that genetic loss of *Abcc6* lead to robust deposition of nanohydroxyapatite within damaged muscle. These findings support a potential role for Abcc6 in preventing aberrant mineralization in skeletal muscle, much akin to its activity in other soft tissues such as the skin, kidney, and cardiovascular system [16, 24, 40–43]. Furthermore, while homozygous null mutations in *Abcc6* have been linked to rare calcification disorders, such as PXE, we observed that even a partial loss (*Abcc6*^+/−^) was sufficient to predispose skeletal muscle to nanohydroxyapatite deposition following injury. While loss-of-function mutations of *Abcc6* such as those seen in PXE are rare (1:50,000), an estimated 1:150-1:300 individuals are carriers for pathologic mutations in *Abcc6* [44]. Our results do not suggest that all individuals with either partial or biallelic pathologic mutation in *Abcc6* will develop HO, but they rather suggest that these individuals may be at risk for the formation nanohydroxyapatite within damaged soft tissue, which may predispose damaged skeletal muscle to ossification if persistent. Furthermore, considering that severely injured patients prone to HO formation have been reported independently to experience both deposits of dystrophic calcification and failure of macrophage function [45–52], these findings together demonstrate the plausibility for this new loss-of-function paradigm of HO.

The molecular mechanisms through which ABCC6 prevents mineralized of soft tissues are not completely understood. Various groups have provided evidence that alteration to either adenosine [53–56] and/or pyrophosphate production [16, 31, 57] is responsible for the aberrant calcification observed in ABCC6-deficient animal models and patients with PXE. While *Abcc6*-deficient animals develop robust skeletal muscle calcification, preliminary studies utilizing animals deficient in either CD73 or CD39, two critical components of adenosine production, demonstrated minimal skeletal calcification skin to wildtype animals following injury (data not shown) (Fig. 4). These findings suggest that reduced adenosine production [58] does not predispose skeletal muscle to nanohydroxyapatite deposition. Therefore, given the numerous reports of reduced circulating PPi levels in patients with PXE and ABCC6-deficient mice [31, 33–35] and recent reports of successful administration of oral PPi to inhibit connective tissue calcification in an ABCC6-deficient murine model [59], these results together suggest that PPi production and its presence in circulation may be responsible for protecting skeletal muscle from calcification following injury. As pyrophosphate levels were not directly measured in this study, further biochemical and enzymatic investigations will be necessary to confirm PPi direct role in protecting skeletal muscle from trauma-induced calcification.

Regardless of the mechanism, the loss of Abcc6 resulted in robust deposition of nanohydroxyapatite in the injured muscle. However, against our hypothesis, the presence of nanohydroxyapatite did not result in HO. Instead, we observed that macrophages resorb the injury-induced nanohydroxyapatite and muscle repair ensues. The essential role of macrophages in this process is highlighted by our experiments in which macrophages were eliminated using liposome-clodronate. In these experiments, not only did the nanohydroxyapatite deposition persist, but ossification ensued.

These findings are transformative in regard to the role of macrophages in HO. The presence of macrophages and inflammation within damaged skeletal muscle has long been observed, both clinically and in basic science investigations. Although debated, currently, macrophages are thought to be a driving factor of HO pathophysiology. For example, in murine models of FOP, it has been demonstrated that depletion of macrophages reduces HO in this model by ~ 50% [60]. Furthermore, in a preclinical model of neurological HO it was observed that ablation of macrophages reduced the size of HO by 90% [61]. Therefore, while macrophages have been demonstrated to be essential for tissue repair, they dichotomously can promote the inflammatory state, as observed in prior HO studies and conditions such as rheumatoid arthritis. Therefore, when we began our investigations, we recognized that macrophages may or may not be beneficial for proper tissue repair following the deposition of nanohydroxyapatite. Through using the same techniques of macrophage ablation of prior HO studies, our results clearly demonstrated that the macrophages stimulated by injury and nanohydroxyapatite deposition function positively to get rid tissues of dystrophic calcification, thereby preventing subsequent HO.

While prior results seem contradictory to our findings, neither of these previous HO models have been demonstrated to progress through a nanohydroxyapatite precursor. Therefore, we propose that the role of macrophages in HO is variable and rather may be dependent upon the environment the macrophages encounter within the damaged tissue. In support of this theory, prior reports have demonstrated that macrophage-mediated regression of calcification is size and composition dependent. If in the nanometer range, macrophages can effectively phagocytose small hydroxyapatite crystals, such as those found in dystrophic calcification lesions, yet, if particles grow beyond 10 µm, they become difficult for a single macrophage to phagocytose [13, 62]. Like size, the composition of calcification greatly impacts the macrophage phenotype and phagocytosis abilities. If the organic components of bone are present (i.e., collage fibrils), macrophage binding and subsequent phagocytosis is inhibited [63]. Moreover, recent investigations by Villa-Bellosta et al. have demonstrated in vitro that macrophages in the presence of high levels of phosphate adopt a M2-like phenotype and expresses elevated anti-mineralization activity dependent upon PPi production [64, 65]. Aligning these findings with our in vivo observations, we propose that when the macrophages encounter nanohydroxyapatite deposits within the injured skeletal muscle, the M2-like macrophages (or reparative macrophages) can respond to this calcium phosphate-rich environment, and adjust their phenotype to promote anti-mineralization activity and clearance of the calcific deposits. To investigate this hypothesis, further investigation into the phenotype of macrophages surrounding the nanohydroxyapatite deposits is warranted. Together, these findings demonstrated that the role of macrophages in HO is potentially more dichotomous than previously believed, and thus caution should be raised when considering therapeutics aimed at inhibiting macrophages for treating HO, prior to determining whether the macrophages is a driver or protector for HO in that particular pathologic state.

Previously, our laboratory demonstrated that plasmin (a powerful reparative protease) like ABCC6 protects skeletal muscle from the formation of dystrophic calcification following injury [36] (Fig. 4). Yet, plasmin has many additional roles during tissue repair, one of which is promotion of macrophage activity and migration. As such, in plasminogen-deficient mice, we previously observed the formation of dystrophic calcification which persisted over 28 DPI and developed in HO. This observation provided the foundation for the theory that dystrophic calcification and HO, rather than being dichotomous pathologies, may rather be part of the same pathologic continuum. However, given plasmin’s variable roles in tissue repair, we necessitated an additional model to isolate specific steps of the pathologic continuum. The studies presented within represent the use of ABCC6 as a directed model to examine the pathologic continuum of dystrophic calcification to HO. Aligning with the results observed in plasmin-deficient animals, utilizing an isolated model, we found that the formation of dystrophic calcification, if persistent due to reduced macrophage-mediated regression, is sufficient to drive HO.

In summary, these foundational studies reveal a potential new paradigm in HO, where persistent nanohydroxyapatite within damaged skeletal muscle as a result of a loss-of-function mutation, in conjunction with a failure of macrophage-mediated resorption, is sufficient to support HO. If found to be clinically valid, this paradigm suggests that rather than being dichotomous pathologies, nanohydroxyapatite formation and HO may be part of the same pathologic continuum, thus providing a novel therapeutic advantage given that nanohydroxyapatite, unlike mature bone, can be resorbed from the damaged tissues. Furthermore, as current treatment regimens for HO, such as prophylactic drugs and radiation therapy, are focused on preventing bone formation, they likewise target physiologic bone leading to adverse effects on bone regeneration and bone health [66–69]. Yet, by placing nanohydroxyapatite and HO on a pathologic continuum, this expands the number of potential pharmacologic strategies available by applying early therapeutic interventions aimed at nanohydroxyapatite in leu of mature bone. Furthermore, early intervention at the nanohydroxyapatite level potentially allows for improved preservation of physiologic bone formation.

## Electronic supplementary material

Below is the link to the electronic supplementary material.

Supplementary material 1 (TIF 112 KB)

Supplementary material 2 (TIF 11109 KB)

Supplementary material 3 (TIF 20369 KB)
