# Supplementary figures and images for: Trauma-Induced Nanohydroxyapatite Deposition in Skeletal Muscle is Sufficient to Drive Heterotopic Ossification
Source: Calcif Tissue Int. 2018 Dec 4;104(4):411–25. doi: 10.1007/s00223-018-0502-5 (PMC6437294; doi:10.1007/s00223-018-0502-5)

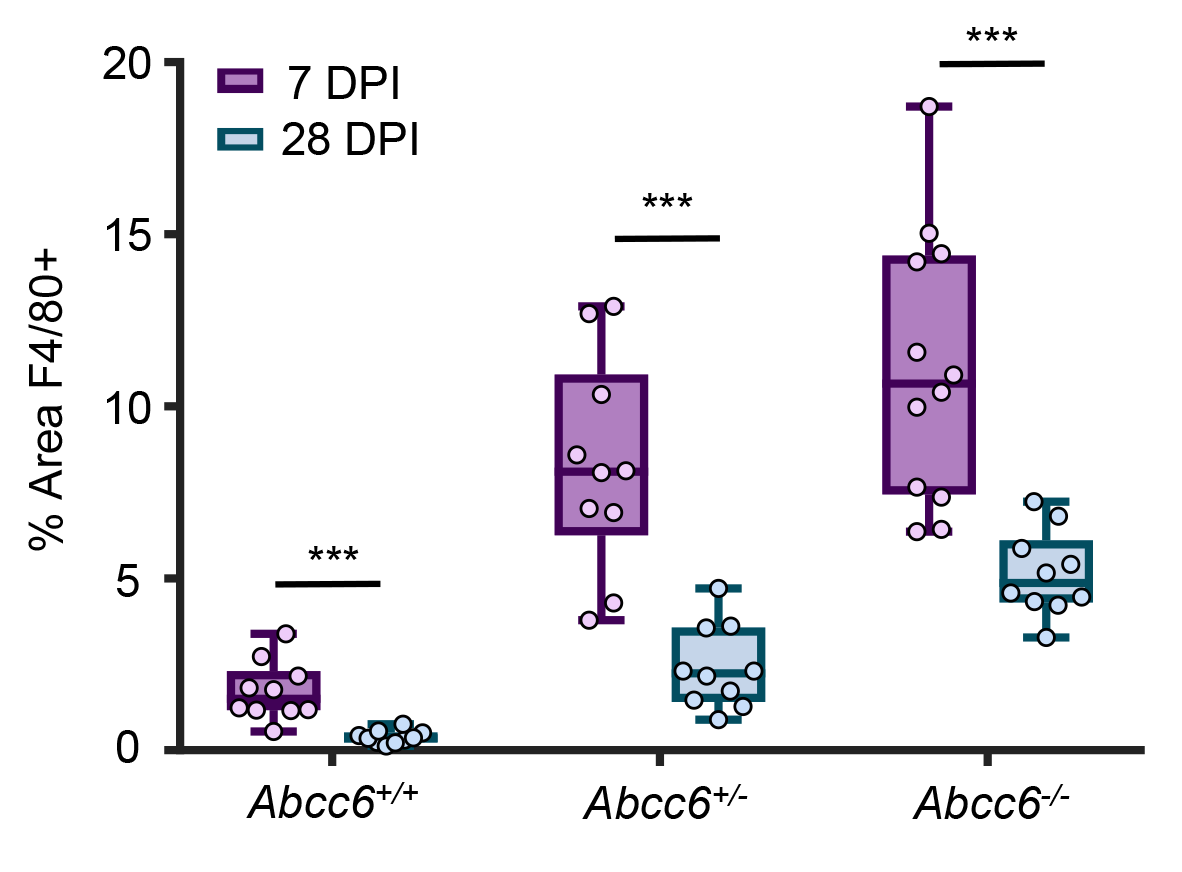

Supplement: Supplementary file 1 — Supplementary material 1 (TIF 112 KB) [file 223_2018_502_MOESM1_ESM.tif]

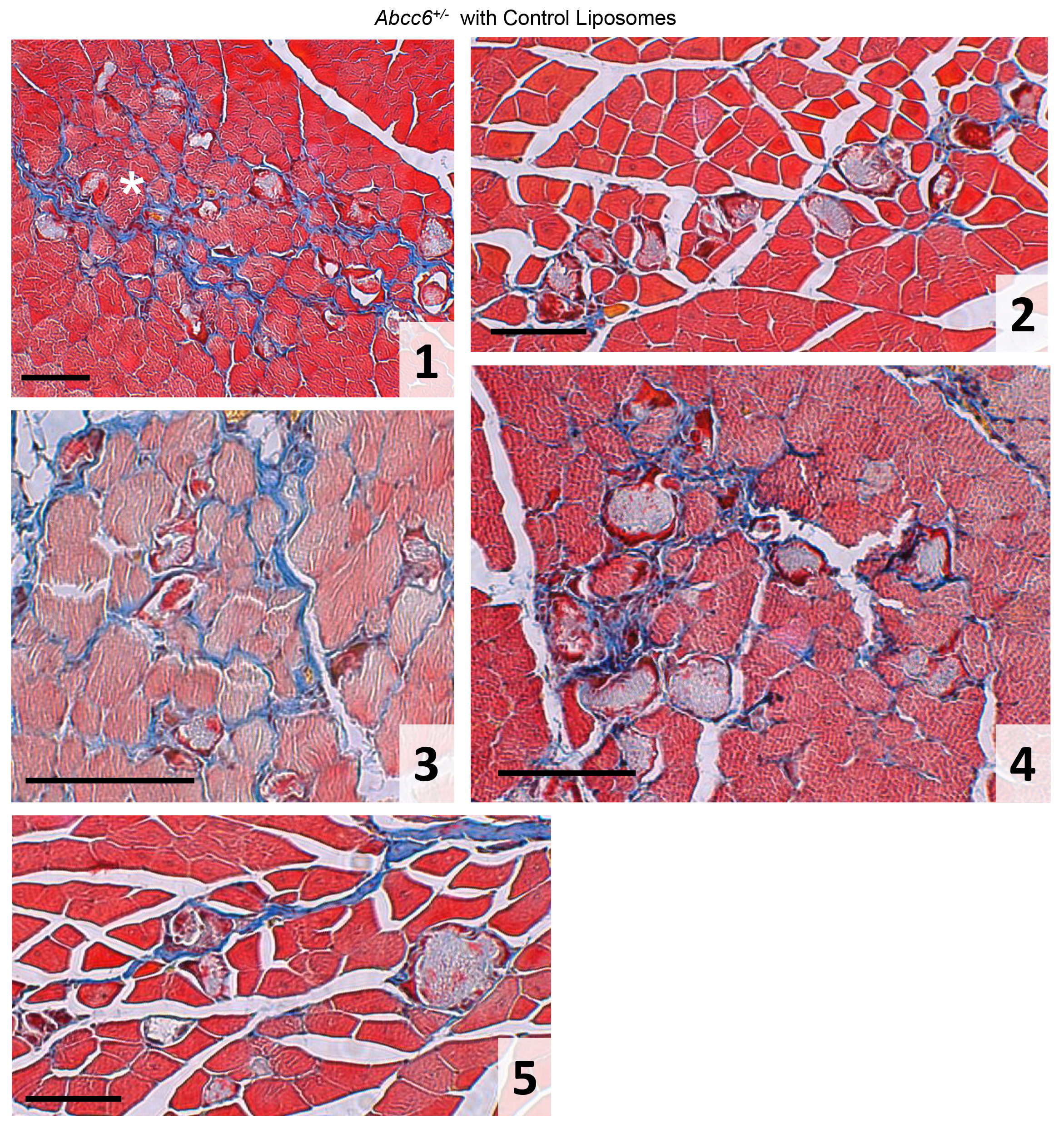

Supplement: Supplementary file 2 — Supplementary material 2 (TIF 11109 KB) [file 223_2018_502_MOESM2_ESM.tif]

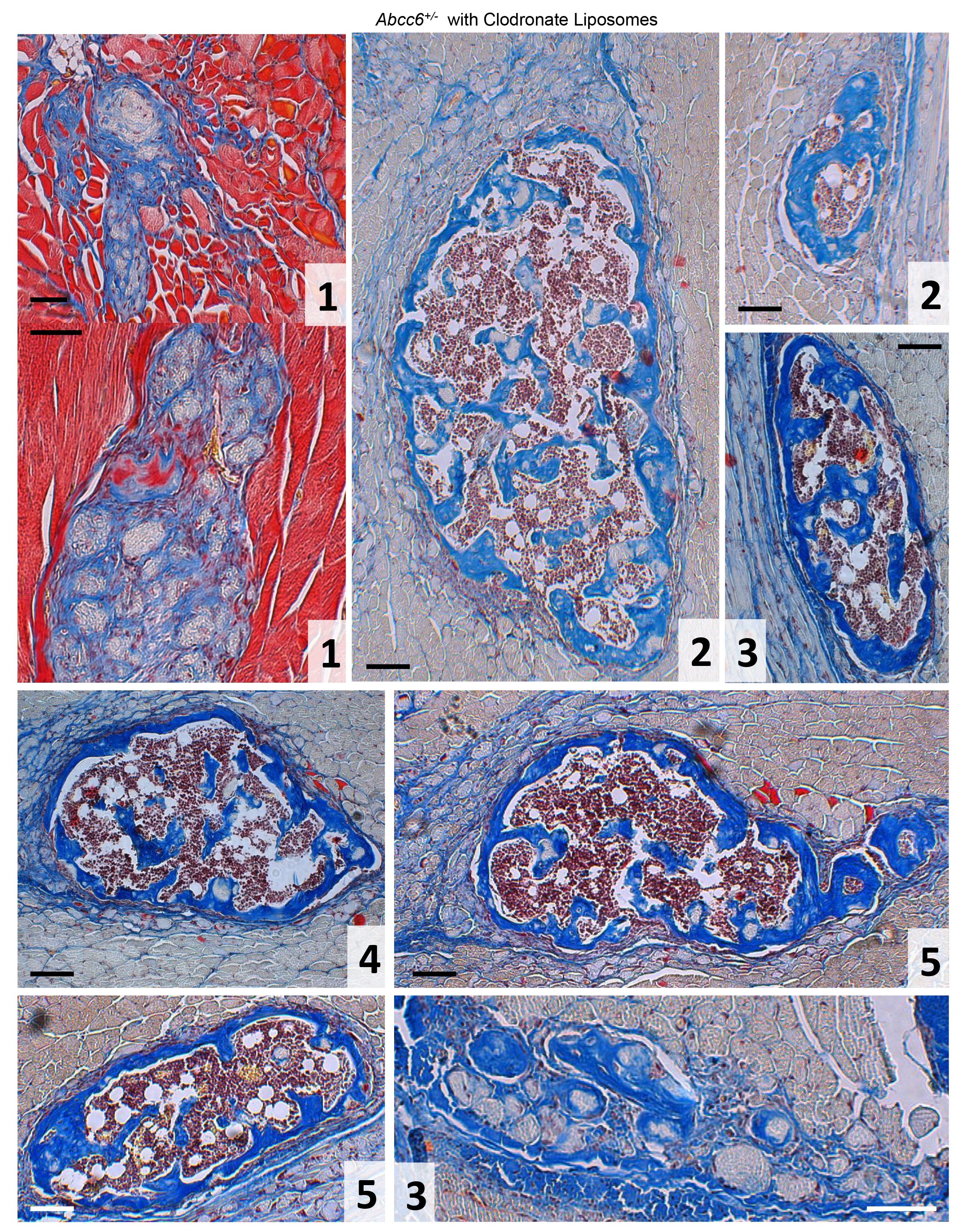

Supplement: Supplementary file 3 — Supplementary material 3 (TIF 20369 KB) [file 223_2018_502_MOESM3_ESM.tif]
